# Optimising perioperative care for hip and knee arthroplasty in South Africa: a Delphi consensus study

**DOI:** 10.1186/s12891-018-2062-2

**Published:** 2018-05-09

**Authors:** U. Plenge, M. B. Nortje, L. C. Marais, J. D. Jordaan, R. Parker, N. van der Westhuizen, J. F. van der Merwe, J. Marais, W. V. September, G. L. Davies, T. Pretorius, C. Solomon, P. Ryan, A. M. Torborg, Z. Farina, R. Smit, C. Cairns, H. Shanahan, S. Sombili, A. Mazibuko, H. R. Hobbs, O. S. Porrill, N. E. Timothy, R. E. Siebritz, C. van der Westhuizen, A. J. Troskie, C. A. Blake, L. A. Gray, T. W. Munting, H. K. S. Steinhaus, P. Rowe, J. G. van der Walt, R. Isaacs Noordien, A. Theron, B. M. Biccard

**Affiliations:** 1 Department of Anaesthesia and Perioperative Medicine, Groote Schuur Hospital and Faculty of Health Sciences, University of Cape Town, Cape Town, South Africa; 2 Department of Orthopaedic Surgery, Groote Schuur Hospital and Faculty of Health Sciences, University of Cape Town, Cape Town, South Africa; 3 Department of Orthopaedic surgery, School of Clinical Medicine, University of KwaZulu-Natal, Pietermaritzburg, South Africa; 4 Department of Orthopaedic Surgery, Tygerberg Medical School, University of Stellenbosch, Cape Town, South Africa; 5 Department Anaesthesia, University of the Free State, Bloemfontein, South Africa; 6 Department of Orthopaedic surgery, University of the Free State, Bloemfontein, South Africa; 7 Department of Physiotherapy, Paarl Provincial Hospital, Paarl, South Africa; 8 Department of Anaesthesia, Paarl Provincial Hospital, Paarl, South Africa; 9 Department of Orthopaedics, Paarl Provincial Hospital, Paarl, South Africa; 10 Arthroplasty and Sports Medicine unit, Department of Orthopaedics, Inkosi Albert Luthuli Central Hospital, University of KwaZulu-Natal, Durban, South Africa; 11 Department of Anaesthesia, Inkosi Albert Luthuli Central Hospital, University of KwaZulu-Natal, Durban, South Africa; 12 Department of Anaesthesia, Critical Care and Pain Management, Grey’s Hospital, Pietermaritzburg, South Africa; 13 Department of Orthopaedic surgery, Grey’s Hospital, Pietermaritzburg, South Africa; 14 Greys Pain clinic, Department of Anaesthesia, Grey’s Hospital, Pietermaritzburg, South Africa; 15 Department of Physiotherapy, Grey’s Hospital, Pietermaritzburg, South Africa; 16 Department of Orthopaedic surgery, Steve Biko Academic Hospital, University of Pretoria, Pretoria, South Africa; 17 Department of Anaesthesia, Steve Biko Academic Hospital, University of Pretoria, Pretoria, South Africa; 18 Department of Physiotherapy, Groote Schuur Hospital and Faculty of Health Sciences, University of Cape Town, Cape Town, South Africa; 19 Department of Anaesthesia, Worcester Hospital, Worcester, South Africa; 20 Department of Orthopaedic Surgery, Worcester Hospital, Worcester, South Africa; 21 Department of Physiotherapy, New Somerset Hospital, Cape Town, South Africa; 22 Department of Orthopaedics, New Somerset Hospital and Christiaan Barnard Memorial Hospital, Cape Town, South Africa; 23 Department of Anaesthesia, New Somerset Hospital, Cape Town, South Africa; 24 Department of Orthopaedic surgery, Victoria Hospital, Cape Town, South Africa; 25 Department of Anaesthesia, Victoria Hospital, Cape Town, South Africa; 26 Department of Physiotherapy, Victoria Hospital, Cape Town, South Africa; 27 Department of Anaesthesiology and Critical Care, Tygerberg Academic Hospital, University of Stellenbosch, Cape Town, South Africa

**Keywords:** Delphi study, Enhanced recovery pathways, Low middle income countries, Total hip and knee arthroplasty, Total hip and knee replacement, Patient outcomes

## Abstract

**Background:**

A structured approach to perioperative patient management based on an enhanced recovery pathway protocol facilitates early recovery and reduces morbidity in high income countries. However, in low- and middle-income countries (LMICs), the feasibility of implementing enhanced recovery pathways and its influence on patient outcomes is scarcely investigated. To inform similar practice in LMICs for total hip and knee arthroplasty, it is necessary to identify potential factors for inclusion in such a programme, appropriate for LMICs.

**Methods:**

Applying a Delphi method, 33 stakeholders (13 arthroplasty surgeons, 12 anaesthetists and 8 physiotherapists) from 10 state hospitals representing 4 South African provinces identified and prioritised i) risk factors associated with poor outcomes, ii) perioperative interventions to improve outcomes and iii) patient and clinical outcomes necessary to benchmark practice for patients scheduled for primary elective unilateral total hip and knee arthroplasty.

**Results:**

Thirty of the thirty-three stakeholders completed the 3 months Delphi study. The first round yielded i) 36 suggestions to preoperative risk factors, ii) 14 (preoperative), 18 (intraoperative) and 23 (postoperative) suggestions to best practices for perioperative interventions to improve outcomes and iii) 25 suggestions to important postsurgical outcomes. These items were prioritised by the group in the consecutive rounds and consensus was reached for the top ten priorities for each category.

**Conclusion:**

The consensus derived risk factors, perioperative interventions and important outcomes will inform the development of a structured, perioperative multidisciplinary enhanced patient care protocol for total hip and knee arthroplasty. It is anticipated that this study will provide the construct necessary for developing pragmatic enhanced care pathways aimed at improving patient outcomes after arthroplasty in LMICs.

## Background

In the past 20 years, enhanced recovery pathways (ERPs) have become increasingly integrated into most surgical fields as standard care in high income countries, as is exemplified by national priority programs [1–3], and the widespread acceptance of the Enhanced Recovery After Surgery (ERAS) society network [4]. ERPs represent a fundamental shift towards a patient-centred, multidisciplinary-driven continuity of care that aim to attenuate surgical stress and expedite recovery [5]. Studies on total joint arthroplasty (TJA) for both hips and knees have shown that implementation of an evidence-based, structured approach to patient care decreases postoperative morbidity and consequently length of stay without increasing readmission rate [6–8].

However, in low- and middle-income countries (LMICs), the value of implementing ERPs is yet to be explored. This may be because: i) the perception that current hospital resources may make it difficult to develop and implement structured and sustainable protocols to enhance postoperative recovery, and ii) short and long-term data collection on the quality of the work provided is scarce, inhibiting the ability to benchmark clinical results and improve the service provided to patients. Despite these challenges, a healthcare system in a middle-income country such as South Africa may benefit from the implementation of ERPs through reduced postoperative morbidity and the associated cost reductions, as has been demonstrated in high-income countries (HICs) [9].

While the goals of implementing ERPs can be expected to be independent of a country’s economic status, we believe the differences in patient demographics, healthcare infrastructure and healthcare resources between HICs and LMICs warrants a LMIC derived programme of enhanced care to facilitate practice change and improve patient outcomes in these settings. The aim of our study was therefore to establish multidisciplinary consensus on; i) preoperative risk factors associated with poor outcomes, ii) perioperative interventions considered necessary to improve outcomes, and iii) important postsurgical patient and clinical outcomes. This study was conducted in South Africa, which represents an upper-middle-income country, as defined by the World Bank [10]. However, as this work was conducted in the public healthcare sector, and South Africa has one of the world’s highest levels of inequality [11], it is likely that this work reflects the state funded healthcare system of a LMIC, as opposed to high-middle-income countries. This assumption is supported by the South African public healthcare service data from the African Surgical Outcomes Study, where the median number of specialists per 100,000 population was 0.9 (IQR 0.2-1.9) (unpublished data) [12], which is well below the recommended 20–40 specialists per 100,000 population [13].

## Methods

We conducted a Delphi survey with experts from different fields involved in the care of arthroplasty surgical patients in South Africa. The Delphi study is an accepted method for achieving convergence of opinions concerning knowledge solicited from experts within specific fields, and has been adopted for priority-setting in medicine [14]. The technique is an iterative process which allows the participant to refine his or her prioritization of items, in an anonymous manner, based on the group’s work from round to round and with controlled feedback of opinions [15].

### Participant recruitment

Participants were recruited from all the hospitals which we knew had a history of performing elective TJAs. This approach was necessary, as currently there is no national arthroplasty database of public hospitals performing TJAs in South Africa. We invited orthopaedic arthroplasty surgeons, anaesthetists and physiotherapists from 18 regional and central hospitals in the public sector covering seven of the nine provinces in South Africa. They were contacted by email and asked to participate in four sequential studies aimed at improving perioperative care for patients scheduled for primary elective unilateral hip and knee TJA in South Africa. The Delphi study is the first of these four studies. For a hospital to participate we required participation of both the Anaesthesia and the Orthopaedic Departments in the project. With the use of telephone calls, face-to-face meetings and further email correspondence, 33 experts in the perioperative management of arthroplasty patients from 10 hospitals representing four provinces accepted the invitation to participate in these four studies. Reasons for exclusion from the study where i) not confirming their participation (5) or ii) declining to participate due to lack of interest or lack of resources to participate in this and future studies (3). Prior to commencement of the Delphi study, the participants were given detailed information of the Delphi process and how consensus would be defined.

### The Delphi process

This Delphi survey was conducted over 3 months from December 2016 to March 2017. In the first round participants submitted suggestions for; i) risk factors associated with poor outcome, ii) best practices for preoperative, intraoperative and postoperative interventions to improve postoperative outcomes and iii) important patient and clinical outcomes to benchmark care, deemed relevant in the South African context for patients scheduled for primary elective unilateral hip and knee TJA. Participants were encouraged to elaborate on how to quantify these components and provide supporting references. UP and BMB grouped the responses in each category into statements. The category statements and supporting references were shared with all participants. In the second Delphi round, the participants were asked to rank the top-ten statements in each category, and where possible, add further comments or relevant references. Based on participants’ responses, statements that overlapped were grouped together prior to the third Delphi round. In the third round the participants were presented with their individual as well as the overall group ranking of the prioritised statements within each category. They were asked to re-evaluate their previous round’s ranking, considering the group ranking and where possible when their rankings differed greatly from that of the group, to add further comments or references supporting their decision. In the fourth and final round, participants were given an opportunity to present any strong disagreement with the priority rankings from the third Delphi round with a Skype teleconference. Non-participation in the fourth round indicated agreement with the proposed Delphi priorities from the third round. Following the teleconference, the consensus of the group was taken as final. UP and BMB were neutral in the prioritization of statements throughout the study.

### Statistical analysis

The rank order of the research priorities for each round was established using a reverse scoring system i.e. a respondent’s rank of 1 received 10 points, down to a rank of 10, which received 1 point. The scores of the respondents were combined for each round to develop the research priority rank order**.**

## Results

### Participants and response rate

The recruited participants included 13 arthroplasty surgeons, 12 anaesthetists and 8 physiotherapists involved in hip and knee arthroplasty. Response rate in the first round was 97% (32/33), 91% (30/33) in the second round and 91% (30/33) in the third round. In the fourth round, all 33 participants accepted the ranking of the prior third Delphi round. However, three participants contributed in the fourth round to a refinement of two of the Delphi statements. The first was an amalgamation of “peripheral nerve blocks” with “multimodal opioid-sparing analgesia regimen” in the postoperative intervention category, which changed the overall ranking in this category. This change clarified that non-opioid analgesic regimens can include regional anaesthesia. The second change was to define “long term survival” in the outcome category as “1-year mortality”, to ensure an objective outcome variable.

### Preoperative risk factors

Two hundred forty-seven suggestions were submitted for round 1 for preoperative risk factors believed to be associated with poor outcomes in patients scheduled for primary elective unilateral hip and knee TJA. The suggestions were categorised into 36 broad statements for round 2 which were refined to 28 statements for round 3. The ten prioritised risk factors identified after the second round did not change in the subsequent rounds (Table 1).

**Table 1:** The ten prioritised preoperative risk factors considered most important determinants of poor outcomes in patients scheduled for primary elective unilateral hip and knee total joint arthroplasty in South Africa

1. Poor general health (ASA 3 and above)	
2. Impaired cardiovascular functional status	
3. Advanced age	
4. Preoperative mobility	
5. Obesity or chronic malnutrition	
6. Recent or current sources of infection (e.g. bladder, respiratory, dental etc.)	
7. Preoperative chronic pain	
7. Matching surgical complexity with surgical experience or skill	
9. Psychiatric disorders and/or cognitive impairment	
10. Preoperative anaemia	

*ASA* American Society of Anesthesiologists

### Preoperative interventions

Round 1 yielded 166 suggestions of preoperative interventions judged to be important to improve outcomes following primary elective unilateral hip and knee TJA. These were amalgamated into 14 statements in round 2 and further refined to 11 different statements for round 3. The ten priorities identified after the second round did not change in subsequent rounds (Table 2).

**Table 2:** The ten prioritised preoperative interventions considered most important determinants to improve outcomes following primary elective unilateral hip and knee total joint arthroplasty in South Africa

1. A patient optimisation clinic	
2. Multidisciplinary planning	
3. Patient education	
4. Infection prevention	
5. Establishing high-volume units	
6. Smoking cessation	
7. Optimisation of preoperative analgesia regimen	
8. Minimise preoperative fasting	
9. Establish a patient blood management programme	
10. Alcohol cessation	

### Intraoperative interventions

One hundred forty-four suggestions for intraoperative interventions believed to improve postoperative outcomes following primary elective unilateral hip and knee TJA were submitted in round 1. These were amalgamated into 18 statements for the second round and further refined to 11 statements for round 3. The ten priorities identified by the second round, did not change in the fourth round (Table 3).

**Table 3:** The ten prioritised intraoperative interventions considered most important determinants to improve outcomes following primary elective unilateral hip and knee total joint arthroplasty in South Africa

1. Meticulous surgical technique	
2. Infection prevention	
3. Optimisation of prosthesis choice and placement	
4. Multimodal opioid-sparing analgesia regimen	
5. Monitoring and optimisation of haemodynamics	
6. Central neuraxial anaesthesia	
7. Establish a patient blood management programme	
8. Temperature regulation	
9. Glycaemic control	
10. Deep vein thrombosis prophylaxis	

### Postoperative interventions

The first Delphi round yielded 181 suggestions of important postoperative interventions to possibly improve outcomes following primary elective unilateral hip and knee TJA. These were amalgamated into 23 statements for the second Delphi round and further refined to 17 statements for the third Delphi round. The final ten priorities were agreed upon in the fourth round of the Delphi process, following amalgamation of “peripheral nerve blocks” into “multimodal opioid-sparing analgesia regimen” (Table 4).

**Table 4:** The ten prioritised postoperative interventions considered most important determinants to improve outcomes following primary elective unilateral hip and knee total joint arthroplasty in South Africa

1. Early mobilisation after surgery	
2. Standardised orthopaedic nursing care	
3. Multimodal opioid-sparing analgesia regimen	
4. Active management of medical co-morbidities	
5. DVT prophylaxis	
6. A pain management team	
7. Patient empowerment in his or her recovery	
8. Patient controlled analgesia	
9. Multidisciplinary ward rounds	
10. Postoperative rehabilitation	

### Important patient and clinical outcomes

One hundred sixty-four suggestions were made in the first Delphi round for important patient and clinical outcomes following primary elective unilateral hip and knee TJA. These were categorised into statements for the second Delphi round and further refined to 23 statements for the third Delphi round. The ten prioritised outcomes did not change after the second round (Table 5).

**Table 5:** The ten prioritised patient and clinical outcomes considered most important following primary elective unilateral hip and knee total joint arthroplasty in South Africa

1. Patient reported outcome measures	
2. Postoperative pain at rest and during movement	
3. 1-year mortality	
4. Early mobilisation	
5. Prosthetic joint infection rate	
6. Joint range of motion	
7. Major adverse cardiac events	
8. Hospital length of stay	
9. Implant longevity	
10. Cost of care	

## Discussion

This study reports a national consensus of the predictors of morbidity, perioperative interventions to improve surgical outcomes, and the clinical outcomes necessary to document perioperative success for patients scheduled for primary elective unilateral hip and knee TJA in South Africa. These findings provide the information necessary to develop a feasible enhanced care programme for South African arthroplasty patients.

The multidisciplinary involvement of regional and central hospitals performing TJAs across South Africa provides a realistic consensus of the factors needed for an enhanced care arthroplasty programme in the public service in South Africa. We believe that the “buy-in” by the participants was high, and this is important for successful organisational change [16]. Furthermore, consensus on the priorities was established early (within Delphi round 2) in four of the five categories, supporting the validity of the final consensus document [17].

However, this study also has limitations. Firstly, while expert consensus is the lowest level of evidence, it is an established method to facilitate clinical guidelines when the evidence is limited [18], particularly when study interventions and study results might not be transferable to settings with a different socio-economic and demographic structure. Furthermore, group consensus studies can expedite the transformation of evidence-based knowledge gained in HICs into practical implementation in LMICs [19], which is why we believe this process is entirely appropriate for the public health service in South Africa, and may be applicable to other LMICs. In our study we have: i) identified feasible interventions which may improve patient outcomes in a resource limited environment, and ii) prioritized which interventions are preferable for implementation if resources do not allow for adoption of all suggested interventions in clinical practice. We believe this approach will allow all sites to focus their resources on developing a pragmatic multidisciplinary programme of enhanced care.

A second limitation is the possibility that we did not invite all sites which performs TJAs in South Africa to participate in the study, as the public health care sector currently does not have a national arthroplasty database. Nevertheless, we succeeded in enrolling both regional and central hospitals from different provinces, which ensured a broad representation of specialists involved in TJAs in South Africa. Finally, we did not include the full spectrum of stakeholders involved in the perioperative management of joint arthroplasty patients or patients themselves. However, we believe our consensus document does represent stakeholders who were not participants in this study, as patient relevant outcomes and parameters important to nursing care, physicians, nutritionists and geriatricians are included (Tables 1, 2, 3, 4 and 5).

Identification of modifiable and non-modifiable risk factors is essential to guide surgical decision making and prepare the patient optimally ensuring safe perioperative care [20]. This is important in a country such as South Africa, which has a medium Human Development Index (HDI)^1^ suggesting a higher risk for perioperative mortality compared to countries with high HDI [21]. Hence, addressing the prioritised preoperative risk factors (Table 1) may improve patient outcomes [22]. Additionally, introducing a best practice protocol in the perioperative period (Tables 2, 3 and 4) aims to provide continuity of care with emphasis on less variability and better quality of service provided [23]. Finally, identifying and standardising procedure specific outcomes facilitates benchmarking, which is crucial to improve the quality of patient care [24]. Only recently have such multinational collaborative efforts been instituted for TJAs to guide future trials towards comparable outcomes [25]. Importantly, this international group of patient partners, orthopaedic surgeons, physical therapists, rheumatologists and methodologists successfully achieved consensus for six core outcome domains; i) pain, ii) function, iii) patient satisfaction, iv) revision, v) adverse events and vi) death, which are all represented in our consensus document (Table 5). While this similarity provides external validity to the work of our Delphi group, it also suggests that aspirations for best patient practice is independent of a country’s income status. However, the novelty of our Delphi study remains with the prioritised preoperative risk factors and perioperative interventions, which we hope will facilitate a pragmatic approach to achieving these postoperative goals in our resource limited settings.

## Conclusion

This national multidisciplinary consensus Delphi study has produced priorities for preoperative risk stratification, perioperative interventions, and outcome assessments necessary for benchmarking, from which a pragmatic enhanced care programme for primary elective unilateral hip and knee TJA in South Africa can be developed. It is anticipated that these priorities may either be applicable or encourage other LMICs to initiate a similar Delphi process. The next phase will involve an audit of current perioperative care addressing the prioritised statements, followed by implementation of the Delphi group’s proposed interventions.

## Abbreviations

ASA: American Society of Anesthesiologists
DVT: Deep vein thrombosis
ERAS: Enhanced recovery after surgery
ERPs: Enhanced recovery pathways
HDI: Human Development Index
HICs: High-income countries
LMICs: Low- and middle-income countries
PBM: Patient blood management
TJA: Total joint arthroplasty

## Footnotes

1 ^*^ Human Development Index (HDI) is an index based on i) life expectancy, ii) education and iii) per capita income indicators, which is used to determine whether a nation is a developed or a developing country.
